# Efficacy and acceptability of the S1P receptor in the treatment of multiple sclerosis: a meta-analysis

**DOI:** 10.1007/s10072-021-05049-w

**Published:** 2021-02-01

**Authors:** Jingyi Tong, Qin Zou, Yongmin Chen, Xiaoping Liao, Rong Chen, Lin Ma, Daqi Zhang, Qifu Li

**Affiliations:** 1 Department of Neurology, The First Affiliated Hospital of Hainan Medical University, Haikou, 570100 People’s Republic of China; 2 Department of Psychology, The First Affiliated Hospital of Hainan Medical University, Haikou, 570100 People’s Republic of China

**Keywords:** Multiple sclerosis, S1P receptors, Treatment efficacy, Treatment acceptability, Network meta-analysis

## Abstract

**Background and objective:**

Sphingosine-1-phosphate (S1P) receptors are extensively used in the treatment of multiple sclerosis (MS). However, the optimal therapeutic role of S1P in MS patients has still remained elusive. This network meta-analysis (NMA) systematically evaluated the efficacy and acceptability of S1P receptors, as disease-modifying drugs, in the treatment of patients with MS, so as to find out the most appropriate therapeutic strategy and provide a reliable basis for the prescription of S1P drugs for patients with MS.

**Methods:**

We conducted a systematic review and NMA to compare the efficacy and acceptability of S1P receptors for treating MS patients. Randomized controlled trials (RCTs), which were published until May 2020, were retrieved from the PubMed, Cochrane Library, Embase, and ClinicalTrials.gov databases. The primary outcome in this study was the treatment efficacy for the S1P receptor for MS patients, in terms of decrease in annualized relapse rate. The secondary outcomes were adverse events leading to discontinuation of a study, such as an unfavorable or unintended sign/symptom. Outcomes were appraised using a random effects model expressed as standardized mean differences (SMDs) and risk ratios (RRs) with 95% confidence intervals (CIs), respectively, and were ranked using surface under the cumulative ranking curve (SUCRA) probabilities for hierarchical clustering of interventions.

**Results:**

A total of 13 RCTS were included, which enrolled 10,554 patients. The results of NMA showed that Fingolimod, Laquinimod, Siponimod, Ozanimod, Amiselimod, and Ponesimod were superior to placebo in terms of reducing the annualized relapse rate of MS patients. Regarding efficacy, the best and worst treatments were Amiselimod (0.4 mg; SUCRA 8.1%) and placebo (SUCRA 90.5%), respectively. As for acceptability, the best and worst interventions were Ozanimod (1 mg; SUCRA 20.4%) and Ponesimod (40 mg; SUCRA 96.0%), respectively. The comparison-adjusted funnel plots of annualized relapse rate and side effects in the included studies revealed that there was no significant funnel plot asymmetry

**Conclusions:**

This NMA indicated that Amiselimod (0.4 mg) is the most effective treatment strategy as a S1P receptor for MS patients. However, the abovementioned findings need to be further confirmed in the next researches.

## Introduction

Multiple sclerosis (MS) is a chronic autoimmune disease of the central nervous system (CNS), in which inflammation, demyelination, and axonal loss occur in even early stages of the disease. Nevertheless, in the majority of patients, MS is characterized by recurrent relapses, followed by complete or partial recovery. Recurrent attacks and disease progression eventually lead to an irreversible neurological damage [1]. Therefore, the aim of MS therapy is to regulate the immune system, control inflammation, reduce recurrence, and attenuate the severity of pain caused by neurological dysfunction. MS patients are strongly advised to undergo long-term diseases-modifying therapies (DMTs). Fingolimod (FTY720) is the first oral treatment for MS. It binds to sphingosine 1-phophate receptors on lymphocytes and via downregulation of the receptor that prevents lymphocyte egress from lymphoid tissues into the circulation. The phosphorylation product FTY-720P can competitively bind to S1PR1 to cause the receptor to entrap, inhibit the outflow of lymphocytes from peripheral lymph nodes, stimulate lymphoid organs, reduce peripheral lymphocytes, and play an immunosuppressive role [2]. Sphingosine-1-phosphate (S1P) receptors are also expressed by a variety of CNS cell types and have been shown to influence cell proliferation, morphology, and migration. The first S1P receptor modulator available on the market is FTY720. However, due to the low selectivity of FTY720, adverse reactions (e.g., bradycardia) occur; therefore, its clinical application has been seriously restricted. In order to attenuate such adverse reactions, scientists have developed S1P receptor modulators with a higher receptor selectivity. Amiselimod, Ozanimod, Ponesimod, and Siponimod are synthetic drugs, which are derived from the precursor Fingolimod with the aim of increasing specificity for selected S1P receptor subtypes compared with the original molecule. The aryl hydrocarbon receptor (AhR) is a ligand-dependent-activated transcriptional factor that regulates the metabolism of xenobiotic and endogenous compounds.

A network meta-analysis (NMA) can be conducted to compare multiple treatments that may not have been compared directly in head-to-head clinical trials. The appropriate use of NMAs can lead to enhanced decision-making in situations where head-to-head clinical trials do not exist; however, decision makers need to be aware of the potential challenges that can arise if NMAs are conducted that do not adequately adjust for cross-trial heterogeneity The present NMA systematically evaluated the efficacy and acceptability of S1P receptors, as disease-modifying drugs, in the treatment of patients with MS, so as to find out the most appropriate therapeutic strategy and provide a reliable basis for the prescription of S1P drugs for patients with MS.

## Materials and methods

### Literature search and study selection

NMA, in the context of a systematic review, is a meta-analysis, in which multiple treatments (that is, three or more) are compared using both direct comparisons of interventions within randomized controlled trials (RCTs) and indirect comparisons across trials based on a common comparator [3]. RCTs, which were published until May 2020, were retrieved from the PubMed, Cochrane Library, Embase, and ClinicalTrials.gov databases. The titles and abstracts of studies retrieved from the search process were read, and we thoroughly reviewed the full texts of relevant articles to determine whether the retrieved studies were eligible to be included in this NMA. Disagreements were resolved by a third reviewer or consensus-based discussion. The search process was carried out using the following search items: [(multiple sclerosis) OR (MS)] AND [(s1p receptor)OR (s1pr) OR (s1pr1) OR (s1pr2) OR (s1pr3) OR (s1pr4) OR (s1pr5)], without any language restrictions. Regarding eligibility, only parallel RCTs were selected, whereas crossover trials, single-arm trials, case reports, and conference papers were excluded. The study population included MS patients who were treated with S1P receptors. However, in the process of literature search, we found that AhR receptor, e.g., Laquinimod, had also been used as a substitute for Fingomod in the treatment of MS. Therefore, we included MS patients who were treated with Laquinimod as a secondary observational indicator to compare the efficacy and acceptability of S1P receptor with AhR receptor.

### Data extraction and quality assessment

Two reviewers independently classified the therapies, extracted the key study parameters through a standardized data abstraction form, and assessed the quality of trials and the risk of bias according to the Cochrane Collaboration’s tool [4]. Disagreements were resolved by a third reviewer or consensus-based discussion. The characteristics of the included studies are summarized in Table 1. After excluding irrelevant studies, it was attempted to further read abstracts and full texts to determine eligible studies. If necessary, we contacted the corresponding authors via email or phone to obtain information required for the NMA. If the standard deviation of the original data could not be calculated, a standard deviation calculator and Cochrane Handbook for Systematic Reviews of Interventions [5] were used. The following data were extracted from each article in the screening process: title, the first author’s full name, year of publication, the corresponding author’s country of origin, assessment of risk of bias, and outcome indicators.

**Table 1:** Characteristics of the included studies

References	Treating arm	Regimen	Number (female/male)	Age (years)	Baseline EDSS score	Treatment duration	Follow-up timing	Dropout	Double-blind
Cohen et al.	Fingolimod (1.25 mg)	1.25 mg, orally, daily	426 (293/133)	35.8±8.4	2.21±1.31	12	12	62	Yes
	Fingolimod (0.5 mg)	0.5 mg, orally, daily	431 (149)	36.7±8.8	2.24±1.33	12	12	44	Yes
	Interferon beta-1a	30 μg, im, weekly	435 (295/140)	36.0±8.3	2.19±1.29	12	12	51	Yes
Kappos et al.	Fingolimod (1.25 mg)	1.25 mg, orally, daily	93 (70/23)	38.3	2.7	12	12	21	Yes
	Fingolimod (0.5 mg)	0.5 mg, orally, daily	92 (65/27)	38.0	2.5	12	12	14	Yes
	Placebo	Placebo, orally, daily	92 (61/31)	37.1	2.6	12	12	19	Yes
Kapoos et al.	Fingolimod (1.25 mg)	1.25 mg, orally, daily	429 (295/134)	37.4±8.9	2.4±1.4	24	24	96	Yes
	Fingolimod (0.5 mg)	0.5 mg, orally, daily	425 (296/126)	36.6±8.8	2.1±1.1	24	24	56	Yes
	Placebo	Placebo, orally, daily	418 ()	37.2±8.9	2.2±1.2	24	24	86	Yes
Calabresi et al.	Fingolimod (1.25 mg)	1.25 mg, orally, daily	370 (281/89)	40.9±8.9	2.5±1.3	24	24	119	Yes
	Fingolimod (0.5 mg)	0.5 mg, orally, daily	358 (275/83)	40.6±8.4	2.4±1.3	24	24	86	Yes
	Placebo	Placebo, orally, daily	355 (288/67)	40.1±8.4	2.4±1.3	24	24	100	Yes
Saida et al.	Fingolimod (1.25 mg)	1.25 mg, orally, daily	57 (39/18)	36.0±9.3	1.8±1.7	6	6	6	Yes
	Fingolimod (0.5 mg)	0.5 mg, orally, daily	57 (40/17)	35.0±9.0	2.3±1.9	6	6	9	Yes
	Placebo	Placebo, orally, daily	57 (39/18)	35.0±8.9	2.1±1.7	6	6	6	Yes
Comi et al.	Laquinimod (0.6 mg)	0.6 mg, orally, daily	106	18-50	2.3±1.1	36	36	6	Yes
	Placebo	Placebo, orally, daily	102	18-50	2.5±1.1	36	36	11	Yes
Comi et al.	Laquinimod (0.6 mg)	0.6 mg, orally, daily	550 (391/159)	38.9±9.2	2.6±1.3	24	24	113	Yes
	Placebo	Placebo, orally, daily	556 (368/188)	38.5±9.1	2.6±1.3	24	24	119	Yes
Vollmer et al.	Laquinimod (0.6 mg)	0.6 mg, orally, daily	434 (282/152)	36.7	2.5	24	24	81	Yes
	Interferon beta-1a	30 μg, im, weekly	447 (321/126)	38.5	2.5	24	24	69	Yes
	Placebo	Placebo, orally, daily	450 (321/129)	37.5	2.5	24	24	91	Yes
Selmaj et al.	Siponimod (2 mg)	2 mg, orally, daily	49 (34/15)	37.4±8.9	2.3±1.1	6	6	5	Yes
	Placebo	Placebo, orally, daily	62 (45/17)	35.4±8.6	2.4±1.2	6	6	3	Yes
Kappos et al.	Siponimod (2 mg)	2 mg, orally, daily	1105 (669/436)	48±7.8	5.4±1.1	24	24	135	Yes
	Placebo	Placebo, orally, daily	546 (323/223)	48.1±7.9	5.4±1.0	24	24	57	Yes
Cohen et al.	Ozanimod (1.0 mg)	1.0 mg, orally, daily	433(291/142)	36.0±8.9	2.6±1.15	24	24	43	Yes
	Ozanimod (0.5 mg)	0.5 mg, orally, daily	439(287/152)	35.4±8.8	2.5±1.17	24	24	39	Yes
	Interferon beta-1a	30 μg, im, weekly	441(304/137)	35.1±9.1	2.5±1.16	24	24	21	Yes
Kappos et al.	Amiselimod (0.4 mg)	0.4 mg, orally, daily	104 (73/71)	37.6±8.7	2.6±1.3	24	24	10	Yes
	Amiselimod (0.2 mg)	0.2 mg, orally, daily	103 (69/36)	38.0±9.6	2.8±1.3	24	24	8	Yes
	Amiselimod (0.1 mg)	0.1 mg, orally, daily	105 (67/36)	37.2±9.4	2.9±1.3	24	24	9	Yes
	Placebo	Placebo, orally, daily	103 (72/32)	37.2±8.5	2.7±1.3	24	24	7	Yes
Olsson et al.	Ponesimod10mg	10 mg, orally, daily	108 (71/37)	36.9±9.2	2.4±1.25	6	6	18	Yes
	Ponesimod20mg	20 mg, orally, daily	114 (77/37)	35.5±8.5	2.2±3.1	6	6	15	Yes
	Ponesimod40mg	40 mg, orally, daily	119 (79/20)	36.5±8.5	2.2±1.17	6	6	25	Yes
	placebo	Placebo, orally, daily	121 (85/36)	36.6±8.6	2.3±1.24	6	6	11	Yes

The primary outcome in this study was the treatment efficacy for S1P receptor for MS patients, in terms of decreased annualized relapse rate (ARR). The secondary outcomes were adverse events leading to discontinuation of a study, such as an unfavorable or unintended sign/symptom.

### Data collection and analysis

The NMA was conducted using Stata 15.0 software (StataCorp LLC, College Station, TX, USA). When dichotomy was applied to outcome measures, risk ratio (RR), and 95% confidence interval (CI) of each outcome indicator were calculated. If the CI does not contain the null hypothesis value, the results are statistically significant. Standardized mean difference (SMD) and 95% CI of each outcome indicator were calculated when the outcome variable was continuous. In the presence of three-arm or more trials, the two arms of all possible combinations were first broken, and a network of evidence for making comparison between various treatments was then established. To do this, we first completed a network of evidence for each outcome measure, in which the size of each node represented the total number of subjects in each intervention, and thickness of each line indicated the number of studies that compared the two interventions. In case of the existence of a network diagram, which is a graphical depiction of the structure of a network of interventions, calculate inconsistency factor (IF), and its 95% CI to evaluate the consistency of each closed-loop. The lower limit of 95% CI is equal to 1, indicating good consistency; otherwise, the closed loop is considered to have obvious inconsistencies. In order to facilitate the process of the interpretation of odds ratio (ORs), the probability of each intervention was computed as the safest or the most satisfactory treatment method of the Bayesian approach based on probability values, and thus summarized as surface under the cumulative ranking curve (SUCRA). A larger SUCRA value symbolized a better rank of intervention. The smaller the SUCRA is, the better the treatment measures will be. Cluster analysis (CA), a multivariate tool used to organize a set of multivariate data (observations, objects) into groups called clusters, was employed to find out the most appropriate intervention measures. A comparison-adjusted funnel plot was developed to indicate whether publication bias would be existed in the ARR and the rate of discontinue due to adverse events. For the evaluation of inconsistency between the direct and indirect comparisons conducted in the eligible studies, we used the design-by-treatment-interaction model, loop-specific approach, and node-splitting model [5, 6].

## Results

### Study characteristics

**Fig. 1:**
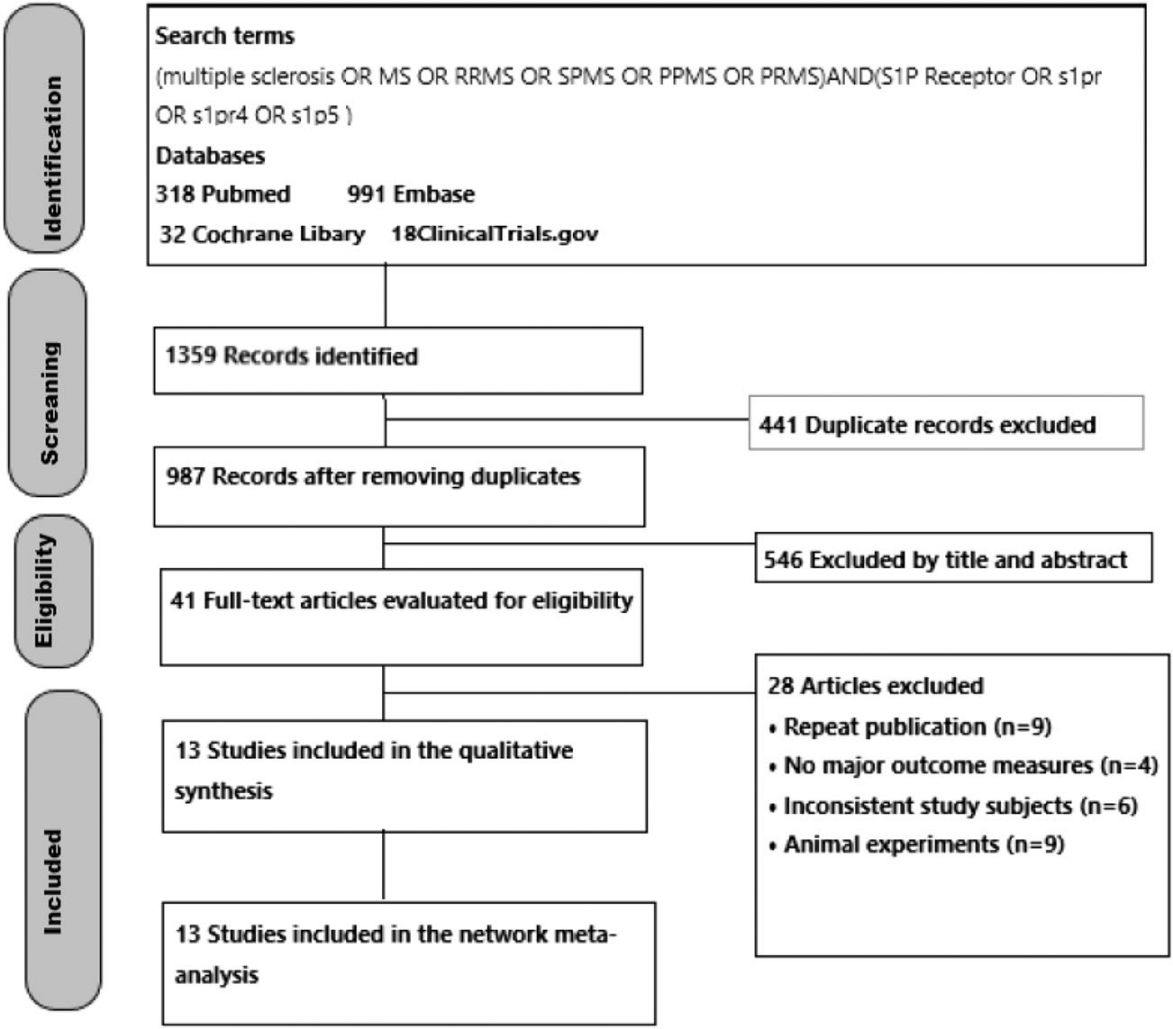
Flowchart of the study selection

**Fig. 2:**
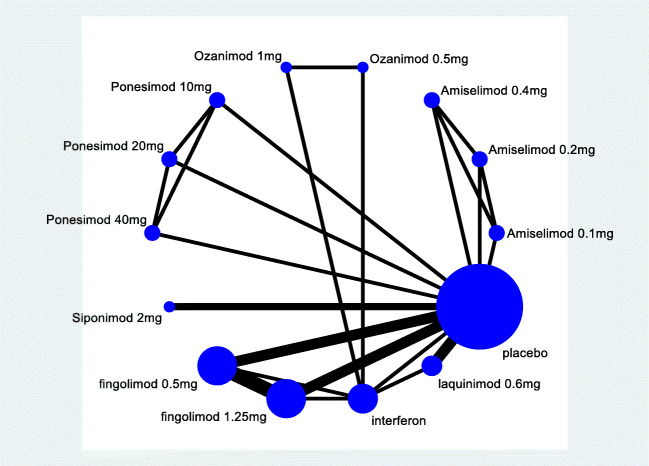
A network diagram representing direct comparisons among treatments. The size of each node indicates the number of randomized allocated participants, and the idth of each line represents the number of trials involved in each comparison

Figure 1 depicts the flowchart of literature search. After retrieving data from electronic databases, we retrieved 1359 articles. After excluding ineligible studies, 13 RCTs were included (5 two-arm studies, 6 three-arm studies, and 2 four-arm studies) [7–19]. These trials enrolled a total of 10554 participants who were randomized to 6 treatment intervention and placebo groups (Fingolimod, *n*=2619; Laquinimod, *n*=1090; Siponimod, *n*=1148; Ozanimod, *n*=872; Amiselimod, *n*=312; and Ponesimod, *n*=337). Figure 2 schematically illustrates the above-mentioned treatments (14 nodes) and comparative arms (24 comparisons) of the included trials for investigating treatment efficacy and acceptability in the network plot of evidence. Among them, 5 trials compared the efficacy of Fingolimod with placebo or other treatment approaches. With the exception of Laquinimod and Siponimod, the efficacy of other four drugs (Fingolimod, Ozanimod, Amiselimod, and Ponesimod) was compared with placebo at different doses.

Table 1 summarizes the information related to study design and study subjects’ characteristics included in the selected RCTs. The RCTs were published between 2008 and 2018 with a sample size that ranged from 49 to 1099 patients per trial. Eligibility criteria included diagnosis of MS (according to the revised (2005) McDonald criteria) with a relapsing-remitting course. Patients who had not undergone previous treatments or who had received disease-modifying agents were found eligible if they received one or more documented relapses 12 months before screening, two or more documented relapses 24 months before screening, one or more documented relapses in a previous year or two or more in previous 2 years. The follow-up lasted for 4–104 weeks.

The side effects included atrioventricular block, leukoencephalopathy, elevated level of alanine aminotransferase (ALT), dyspnea, and infection. Treatment was terminated as critical side effects were reported in each study.

### Network meta-analysis

The results of treatment efficacy and acceptability are presented in Table 2. Regarding the treatment efficacy, it was revealed that all the interventions were significantly more beneficial than placebo (Fingolimod [(1.25 mg) (SMD, 0.80; 95% CI 0.76–0.84)], Fingolimod [(0.5 mg) (SMD, 0.8; 95% CI: 0.76–0.84)], Interferon [(SMD, 0.92; 95% CI 0.87–0.97)], Laquinimod [(0.6 mg) (SMD, 0.92; 95% CI 0.88–0.97)], Siponimod [(2 mg) (SMD, 0.91; 95% CI 0.87–0.95)], Ozanimod [(1 mg) (SMD, 0.82; 95% CI 0.76-0.89)], Ozanimod [(0.5 mg) (SMD, 0.87; 95% CI 0.80–.94)], Amiselimod [(0.4 mg) (SMD, 0.71; 95% CI 0.59–0.86)], Amiselimod [(0.2 mg) (SMD, 0.95; 95% CI 0.61–1.35)], Amiselimod [(0.1 mg) (SMD, 1.0; 95% CI 0.74–1.34)], Ponesimod [(10 mg) (SMD, 0.82; 95% CI 0.6.3–1.08)], Ponesimod [(20 mg) (SMD, 0.90; 95% CI 0.68–1.19)], Ponesimod [(40 mg) (SMD, 0.76; 95% CI 0.59–0.98)). Amiselimod (0.4 mg) caused the greatest reduction of ARR compared with placebo (Amiselimod (0.4 mg) versus placebo: MD, 0.71; 95% CI 0.59 0.86). Ponesimod (40 mg) was noted as the second most beneficial intervention (MD, 0.76; 95% CI 0.59 0.98). For treatment acceptability, side effects were less likely to occur in MS patients who received Ozanimod (Ozanimod (1 mg) versus placebo: RR, 0.81; 95% CI (0.28, 2.33)), while those side effects were more frequently observed after administration of Ponesimod [(40 mg) (RR, 12.69; 95% CI 1.51–106.36)] and Fingolimod [(1.25 mg) (RR; 2.25, 95% CI (1.49, 3.39)]. In terms of side effects that caused disruption of the trials, Fingolimod (1.25 mg) compared with placebo (RR, 2.25; 95% CI: 1.49–3.39) and Ponesimod (40 mg) compared with placebo (RR, 12.69; 95% CI 1.51–106.36) showed statistical significance, indicating their improper treatment acceptability. The side effects of other drugs (Fingolimod [(0.5 mg) (RR, 1.45; 95% CI 0.94–2.24)], Interferon (RR, 1.12; 95% CI 0.64–1.97), Laquinimod [(0.6 mg) (RR, 1.2; 95% CI 0.58–2.50)], Siponimod [(2 mg) (RR, 1.7; 95% CI 0.8–3.61)], Ozanimod [(1 mg) (RR, 0.81; 95% CI 0.81–2.23)], Ozanimod [(0.5 mg) (RR, 0.87; 95% CI 0.31–2.45)], Amiselimod [(0.4 mg) (RR, 1.79; 95% CI 0.46–6.95)], Amiselimod [(0.2 mg) (RR, 1; 95% CI 0.22–4.49)], Amiselimod [(0.1 mg) (RR, 1.24; 95% CI 0.29–5.21)], Ponesimod [(10 mg) (RR, 5.83; 95% CI 0.6.3–53.76)], Ponesimod [(20 mg) (RR, 5.50; 95% CI 0.60–50.77)]) were not statistically significant in terms of disrupting the trial, demonstrating their proper treatment acceptability.

**Table 2:**
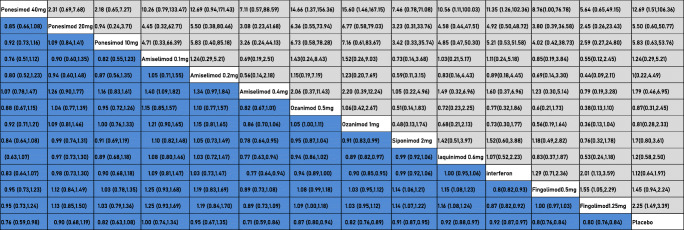
Comparing effect size between treatment groups in the NMA regarding efficacy (lower triangle) and acceptability (upper triangle)

Efficacy (light blue): the result of each cell is the outcome of comparing a drug in the vertical cell with a drug in the horizontal cell (MD (95% CI)). Acceptability (gray): the result of each cell is the outcome of comparing a drug in the horizontal cell with a drug in the vertical cell (RR (95% CI)). Bolded values indicate a drug-based comparison with placebo

**Fig. 3:**
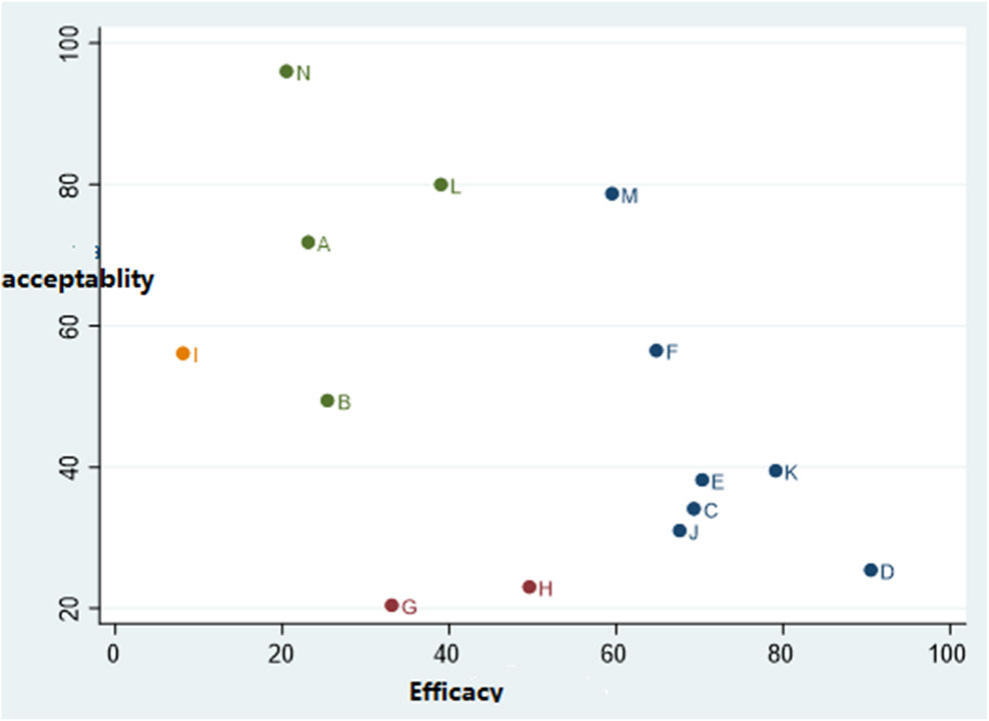
Two-dimensional graphs showing the values of SUCRA for efficacy and acceptability of the S1P receptor used in the treatment of multiple sclerosis. X-axis: EARR; Y-axis: serious adverse events leading to discontinuation of trials (A = Fingolimod (1.25 mg), B = Fingolimod (0.5 mg), C = Interferon, D = Placebo, E = Laquinimod (0.6 mg), F = Siponimod (2 mg), G = Ozanimod (1 mg), H = Ozanimod (0.5 mg), I = Amiselimod (0.4 mg), J = Amiselimod (0.4 mg), K = Amiselimod (0.1 mg), L = Ponesimod (10 mg), M = Ponesimod (20 mg), N = Ponesimod (40 mg))

Furthermore, Fig. 3 indicates the likelihood of the most appropriate intervention with minimum side effects for the included treatments, which is Amiselimod (0.4 mg). Figure 3 shows the relationship between the SUCRA values of efficacy and acceptability in all the studies. Regarding efficacy, the best and worst treatments were Amiselimod (0.4 mg; SUCRA 8.1%) and placebo (SUCRA 90.5%), respectively. As for acceptability, the best and worst interventions were Ozanimod (1 mg; SUCRA 20.4%) and Ponesimod (40 mg; SUCRA 96.0%), respectively.

**Fig. 4:**
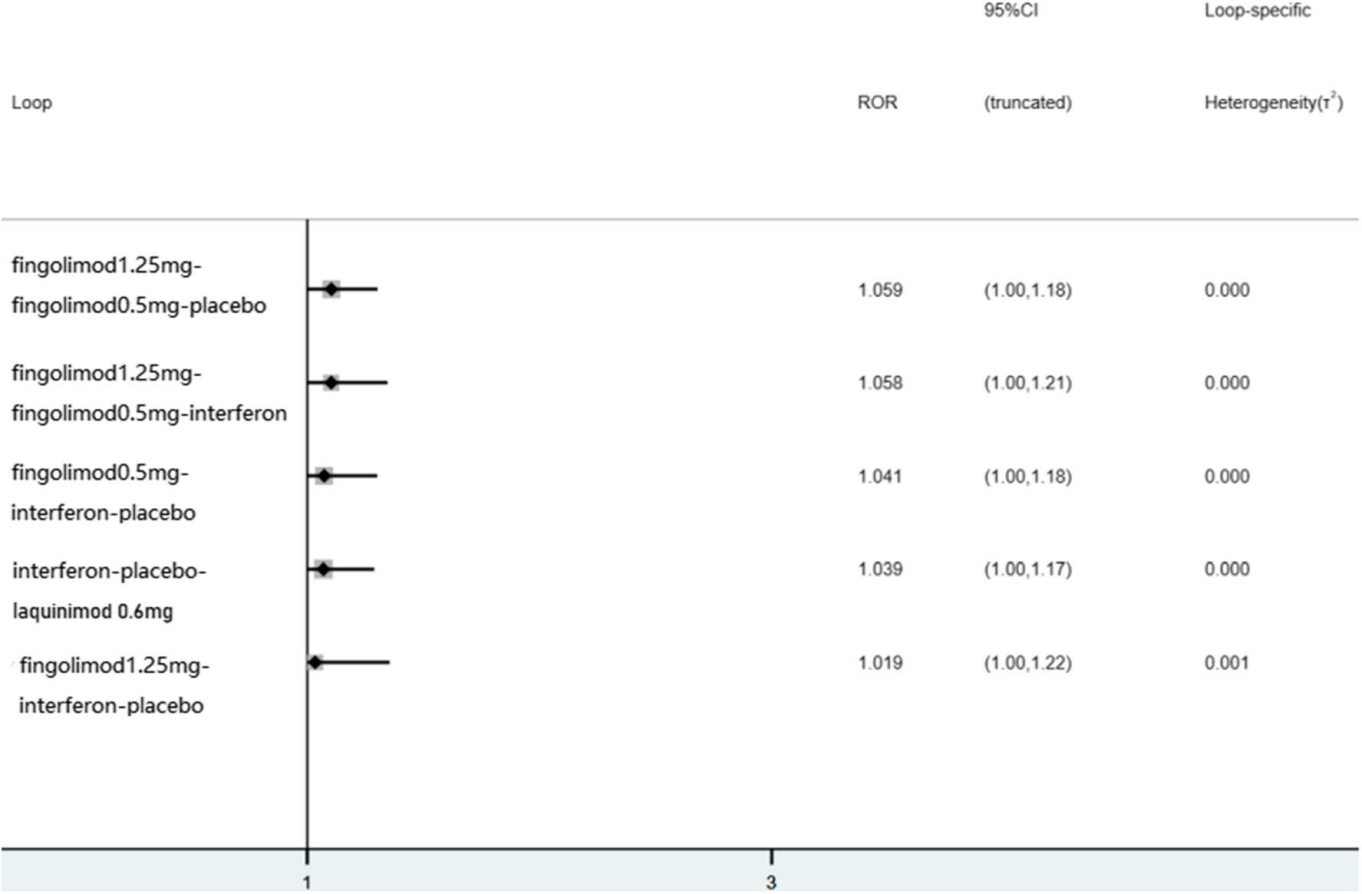
A graph for checking inconsistency. If there would be a closed-loop structure in the evidence network diagram, the inconsistency factor and its 95% CI indicate the consistency of each closed-loop (the lower limit of 95% CI is equal to 1, indicating good consistency; otherwise, the closed loop is considered to have obvious inconsistencies)

**Fig. 5:**
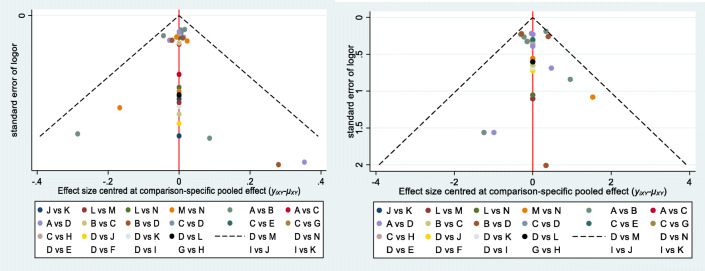
Comparison-adjusted funnel plots. The annualized relapse rate (left) and serious adverse events lead to discontinuation of study (right) were distributed symmetrically at each study site, suggesting the low likelihood of publication bias

We further evaluated the inconsistency of these outcomes using the design-by-treatment, loop-specific approach, and node-splitting model, and found no evidence of statistical inconsistencies (Fig. 4). It should be noted that if the CI covers zero, the coefficient is deemed insignificant (or inconsistent). Figure 5 illustrates the comparison-adjusted funnel plots of ARR and side effects in the included studies, which revealed no significant funnel plot asymmetry, and no evidence of publication bias.

## Discussion

MS is categorized as a rare disease with an estimated 30,000 MS patients in China. Due to the pathological changes of the autoimmune system, the nerve myelin sheath is damaged and peeled, resulting in the impairment of the spinal cord, brain, and optic nerve’s functions. It is an inflammatory demyelinating CNS disease frequently appearing in young adulthood. Supported by an experimental evidence, MS is generally considered a predominantly T cell-mediated autoimmune disease. A typical early disease course is characterized by clinical relapses followed by symptomatic improvement or remissions with an indolent accumulation of symptoms later in life, likely in part due to a component of neurodegeneration as well as ongoing chronic inflammation [20].

S1PR1 is located on lymphocytes, neural cells, endothelial cells, smooth muscle cells, and atrial myocytes, as well as in the atrioventricular node and the conduction system. Functions include the egress of lymphocytes from lymph nodes, neuron migration and function, endothelial permeability, vasculature formation, in addition to the decreased heart conduction. S1PR2 is located in the CNS and on endothelial and smooth muscle cells, influencing hearing and balance function, as well as endothelial permeability and vascular tone. S1PR3 is located on neural cells, atrioventricular node, and the conduction system, as well as on smooth muscle cells. Its functions comprise neural cell migration and function, slowed cardiac conduction, and endothelial permeability. S1PR4 is located solely on lymphocytes, influencing lymphoid tissue expression, as well as modulation of dendritic cells and TH17 cells. S1PR5 is located in the CNS. S1P binds to G-protein-coupled receptors (S1P1–S1P5) to modulate a wide range of physiological systems [21].

To date, several multi-center, double-blinded, randomized, controlled trials have been carried out on S1P receptor. However, the lack of head-to-head comparisons has made it impossible to compare the efficacy and acceptability of s1PR. In the present NMA, the efficacy and acceptability of the interventions were ranked by indirect comparison among studies with an insufficient evidence: a total of 13 RCTs and a total of 10,554 patients were herein included. Compared with placebo, S1P receptors, such as Fingolimod, Siponimod, Ozanimod, Amiselimod, Ponesimod, and Laquinimod could significantly reduce the ARR and disability progression. According to the results of this NMA, Amiselimod (40 mg) possessed the highest efficacy, followed by Ponesimod (40 mg), and Laquinimod had the worst efficacy. In terms of safety, Fingolimod (1.25 mg) and Ponesimod (40 mg) showed a great number of dropouts due to severe adverse reactions compared with placebo, while other drugs did not cause significant adverse reactions compared with placebo. Ozanimod (1 mg) is a drug with the lowest risk of serious adverse reactions, leading to trial discontinuation. It was revealed that Amiselimod (40 mg) possessed the highest treatment efficacy with the least side effects.

Amiselimod was designed as a prodrug S1P receptor modulator lacking S1P3 agonism to avoid bradycardia with the first dose. It is converted to its active metabolite, and it functions as a highly selective S1P1 functional antagonist without S1P3 activity. In addition to its lack of S1P3 agonist activity, it was designed to be converted to the active metabolite in human cells (*in vitro*) more slowly than Fingolimod, because it was thought that its effect on the heart rate of humans may be ameliorated by a gradual increase in concentration of its active metabolite in the heart following administration. Amiselimod-P could result in an approximately five-fold weaker activation of GIRK channels in human primary atrial myocytes compared with Fingolimod-P. Because the S1P1 agonist activity of Amiselimod-P was reported similar to that of Fingolimod-P, the potential for bradycardia caused by Amiselimod-P is likely to be less than that for Fingolimod-P, owing to weaker activation of GIRK channels [22].

Fingolimod acts as a functional antagonist by rapid S1PR1 desensitization, degradation, and internalization in T and B lymphocytes. Investigations with the assistance of the experimental autoimmune encephalomyelitis model (EAE) indicated a higher concentration of Fingolimod in the CNS compared with peripheral compartment, accumulating in white matter and myelin sheaths plays a significant role in modulation of several processes in the CNS, including maturation, proliferation, and migration of neuronal cells that interact and balance brain damage and repair. Although Fingolimod primarily binds to S1PR1, and the other S1P receptors are also affected by Fingolimod therapy. Based on the natural distribution pattern of the S1P-receptor subtypes, especially on atrial myocytes, the risk of cardiac events has notably increased [23].

Ozanimod does not require phosphorylation for activation and it induces a rapid, dose-dependent, and reversible selective reduction. It is an agonist of the S1PR1 and S1PR5 with a 27-fold selectivity for S1PR1 over S1PR5. Its effects can be further mediated by S1PR1 receptor internalization and subsequent ubiquitin-proteasome-dependent degradation, thereby preventing receptor reinstallation in the cellular membrane. Ozanimod binding to S1PR5 can activate specific cells in the CNS, promote myelin regeneration, and prevent synaptic defects, ultimately preventing nerve damage. Ozanimod possesses an excellent advantage in terms of reducing the ARR due to the combination of the two mechanisms “damage reduction + repair enhancement” [24].

Siponimod influences both peripheral B and T cells with pronounced effect on CD4 T cells compared with CD8 T cells, as well as a preferential decline in CD4 naive cells and CD4 central memory T cells (TCM, CCR7+) with less effect on CD4 peripheral effector memory T cells (TPEM, CCR7-). Siponimod decreases oligodendocyte and axonal loss, suggesting that it might be able to protect axons during both the acute and the chronic demyelinating phases of MS. There was no significant effect on remyelination [25]. A meta-analysis reported that topline results from the phase 3 CONCERTO trial showed that the oral treatment for relapsing-remitting multiple sclerosis (RRMS) did not meet its primary endpoint, according to an announcement from the manufacturers [26].

Ponesimod, an iminothiazolidinone derivative, is a reversible, orally active, selective S1P1 modulator. In contrast to Fingolimod, which is a structural analogue of sphingosine, Ponesimod is selective for S1P1; *in vitro*, Ponesimod is at least 10-fold more potent on the S1P1 receptor than on other S1P receptor subtypes. Lymphocytes migrate from the lymph node into blood following an S1P gradient that is maintained by the high levels of S1P in the blood and lymph, which far exceed those at tissues. Binding of Ponesimod to the S1P1 receptor results in rapid and efficient receptor internalization, degradation, and functional antagonism, thereby causing lymphocyte sequestration in the lymph nodes [25].

The present NMA contains a number of limitations. First, the majority of the included studies did not explicitly report random methods, allocation, and concealment schemes and blind methods, and there was possibility likelihood of selective bias, implementation bias, and measurement bias. Second, the research outcome indicator reports do not have a unified standard. For instance, when ARR was reported, the affiliated data were partly expressed as mean ± 95% CI, and some as mean ± standard error of the mean (SEM). Third, in the presence of some factors, e.g., China’s economic and social conditions, several RCTs on administration of drugs have been conducted in China, while further multi-center, long-term, and double-blinded studies are warranted. It should be noted that the majority of studies included in this NMA were conducted in European counties and in the USA, and there may be a significant difference in treatment efficacy between the European and Asian populations.

In summary, S1P receptors are effective in terms of reducing the ARR in patients with MS. Comparing the efficacy and safety of some therapies for MS patients showed that Amiselimod (40 mg) possesses a promising efficacy in terms of reducing the ARR and a low adverse reaction rate. Comparably, Fingolimod possesses satisfactory therapeutic effects, while it has a higher adverse reaction rate. However, the abovementioned conclusions need to be further confirmed in the next researches.
